# Renal Denervation Findings on Cardiac and Renal Fibrosis in Rats with Isoproterenol Induced Cardiomyopathy

**DOI:** 10.1038/srep18582

**Published:** 2015-12-22

**Authors:** Qian Liu, Qi Zhang, Kai Wang, Shengchan Wang, Dasheng Lu, Zhenzhen Li, Jie Geng, Ping Fang, Ying Wang, Qijun Shan

**Affiliations:** 1 Department of Cardiology, The First Affiliated Hospital of Nanjing Medical University.

## Abstract

Cardio-renal fibrosis plays key roles in heart failure and chronic kidney disease. We sought to determine the effects of renal denervation (RDN) on cardiac and renal fibrosis in rats with isoproterenol induced cardiomyopathy. Sixty male Sprague Dawley rats were randomly assigned to Control (n = 10) and isoproterenol (ISO)-induced cardiomyopathy group (n = 50). At week 5, 31 survival ISO-induced cardiomyopathy rats were randomized to RDN (n = 15) and Sham group (n = 16). Compared with Control group, ejection fraction was decreased, diastolic interventricular septal thickness and left atrial dimension were increased in ISO-induced cardiomyopathy group at 5 week. After 10 weeks, cardio-renal pathophysiologic results demonstrated that the collagen volume fraction of left atrio-ventricular and kidney tissues reduced significantly in RDN group compared with Sham group. Moreover the pro-fibrosis factors (TGF-β1, MMP2 and Collagen I), inflammatory cytokines (CRP and TNF-α), and collagen synthesis biomarkers (PICP, PINP and PIIINP) concentration significantly decreased in RDN group. Compared with Sham group, RDN group showed that release of noradrenaline and aldosterone were reduced, angiotensin-converting enzyme (ACE)/angiotensin II (Ang II)/angiotensin II type-1 receptor (AT_1_R) axis was downregulated. Meanwhile, angiotensin-converting enzyme 2 (ACE2)/angiotensin-1-7 (Ang-(1-7))/mas receptor (Mas-R) axis was upregulated. RDN inhibits cardio-renal fibrogenesis through multiple pathways, including reducing SNS over-activity, rebalancing RAAS axis.

Cardiac and renal fibrosis is the result of various cardiovascular injuries. Vice versa, cardio-renal fibrosis promotes heart and kidney disease progression^1^. Both left atrium (LA) enlargement and LA fibrosis play key roles in developing and sustaining atrial fibrillation^2^,^3^. Atrial fibrosis is an independent predictive factor for heart failure, stroke and cardiac death. Ventricular fibrosis leads to left ventricular (LV) dysfunction, myocardial stiffness, ventricular arrhythmia and sudden death^4^,^5^. Previous evidences also showed that renal fibrosis had a strong correlation with chronic kidney disease (CKD) development^6^,^7^. Renal fibrosis leads to kidney failure, hypertension, anemia and electrolyte disturbance. Thus, anti-fibrosis will be a target therapy for cardiovascular disease in the future.

Renal denervation (RDN) is a novel therapy method for patients with resistant hypertension^8^, heart failure^9^, atrial fibrillation^10^, ventricular tachyarrhythmias^11^ and CKD^12^. These diseases were highly associated with organic fibrosis. The chronic over-activation of sympathetic nerve system (SNS) and renin–angiotensin–aldosterone system (RAAS) are central links between organic fibrosis and cardiovascular disease^13^,^14^,^15^. In the present study, we assumed that RDN could directly inhibit cardio-renal fibrosis through rebalancing RAAS (ACE/Ang II/AT_1_R & ACE2/Ang-(1-7)/Mas-R) and decreasing SNS activity. Thus, in order to investigate the effects of RDN on cardio-renal fibrosis, we used a rat model of ISO-induced cardiomyopathy, which imitates chronic over-activity status of SNS and RAAS.

## Methods

### Animals and Experimental Design

All procedures in this study were performed in accordance with the Guide for the Care and Use of Laboratory Animals (National Institutes of Health publication 8th edition, 2011) and were approved by the Nanjing Medical University Experimental Animal Care and Use Committee. The experiment was performed in male Sprague-Dawley rats weighing 180–220 g (Nanjing Medical University Laboratory Animal Center), caged individually at controlled temperature and humidity with a 12-hour light/dark cycle. Echocardiography was performed at baseline, week 5 and 10. ISO injection was performed after echocardiography at baseline. RDN or sham procedure was performed after echocardiography work-up at week 5. At the end of the study, after the third echocardiography work-up and blood collection, all animals were euthanized with an overdose of pentobarbital sodium (200 mg/kg) by intraperitoneal injection.

### ISO-Induced Cardiomyopathy

Sixty male Sprague Dawley rats were randomly assigned to two groups: Control group (n = 10) and ISO-induced cardiomyopathy group (n = 50). Rats in ISO-induced cardiomyopathy group were intraperitoneally injected with 5 mg/kg/d isoproterenol hydrochloride (Sigma, Switzerland)^16^, dissolved in normal saline, once a day for the first 5 consecutive weeks.

### Renal Denervation

After 5 weeks intraperitoneal injection of ISO, 31 survival rats in ISO-induced cardiomyopathy group were divided into 2 groups: RDN (n = 15) and Sham (n = 16) group. With pentobarbital sodium (60 mg/kg intraperitoneal injection) anesthesia, bilateral renal denervation was performed in RDN group, whereas sham RDN procedure was performed in Sham and Control group. RDN was implemented as described previously^17^. Visible nerves along the renal arteries and veins were stripped and picked with 10× magnification. Chemical denervation was conducted by daubing the bilateral renal artery with 20% phenol solution in absolute alcohol. Then the arteries and veins were washed with isotonic saline. For sham RDN procedure, the operation was the same, but the renal arteries and veins were not isolated and the nerves were left intact.

### Echocardiography

Cardiac structure and function were evaluated by echocardiography with Vevo2100-a high resolution imaging system (VisualSonics, Canada) with a MS-250, 16.0–21.0 MHZ imaging transducer at baseline, week 5 and 10. All of the rats were anesthetized by aether before the process of echocardiography work-up.

### Histopathology

After perfusion with ice-cold PBS, the heart and kidney were cut and fixed in 4% phosphate buffered formalin for 48–72 h at 4 °C, then tissues were dehydrated and embedded in paraffin. Apex cordis was fast frozen by liquid nitrogen, then moved to −80 °C. Masson’s trichrome staining was performed to detect cardiac (including left atrium and ventricular) and kidney (including tubulointerstitial, renal perivascular and renal glomerular) fibrosis. Five fields of each sample were randomly selected and collagen volume fraction (CVF) was assessed by Image-Pro Plus 6.0.

### Western Blot

Protein expressions of ACE (AbcamInc, UK), ACE2 (AbcamInc, UK), AT_1_R (AbcamInc, UK), Mas-Receptor (Mas-R) (Alomone Labs, Jerusalem, Israel), Transforming growth factor-β1 (TGFβ1) (R&D Systems, Minneapolis, MN), matrix metalloproteinase 2 (MMP2) (Santa cruz, USA), Collagen I (Santa cruz, USA) and GAPDH (Bioworld, USA) were assessed by western blot technique. All antibodies were applied according to the manufacturer’s instructions. The dilution ratios of each antibody were as follow: ACE, 90kDa, 1/100; ACE2, 92kDa, 1/5000; AT_1_R, 41kDa, 1/800; Mas-R, 1/200, 50kDa; TGF-β1, 1/500, 25kDa; MMP2, 1/200, 55kDa and Collagen I, 1/200, 90kDa.

### ELISA

Blood samples were obtained by EDTA-tubes, and then centrifuged at 3000 rpm at 4 °C for 10 minutes to separate the plasma which stored at −80 °C for later use. Plasma B-type natriuretic peptide (BNP), norepinephrine (NE), aldosterone (ALD), Ang II and Ang-(1-7), tumor necrosis factor α (TNF-α), C-reactive protein (CRP), amino-terminal pro-peptides of types I and III collagen (PINP, PIIINP), and carboxyl-terminal pro-peptide of type I collagen (PICP) levels at week 10 were measured using enzyme linked immunosorbent assay (ELISA) kits, according to the manufacturer’s instructions (Uscn Life Science Inc, Wuhan, China).

### Statistics

Data are expressed as mean ± SEM and analyzed by SPSS 16.0 (SPSS Inc, Chicago, IL, USA). For two groups comparison, data were analyzed with two-tailed unpaired t tests. For multiple groups comparisons, data were performed using one-way ANOVA with LSD test. P < 0.05 was considered statistically significant.

## Results

### Cardiac Structure and Function

**Figure 1:**
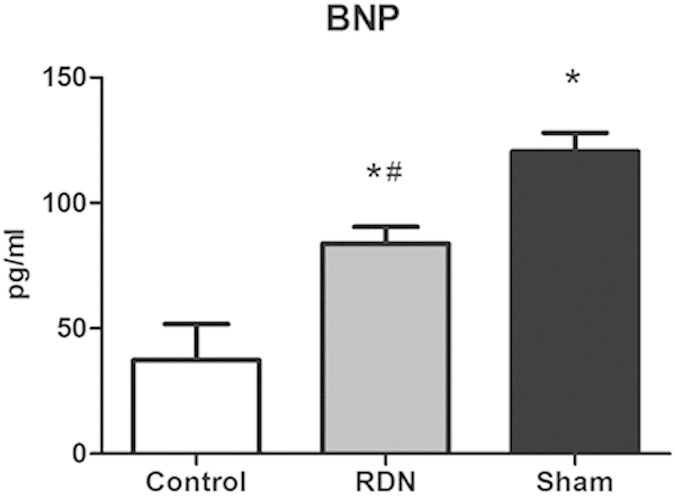
RDN Significantly Reduced BNP Concentration. *P < 0.05, RDN or Sham vs. Control; #P < 0.05, RDN group vs. Sham group (n = 6 per group). BNP, B-type natriuretic peptide.

At week 5, echocardiography revealed significant increases in diastolic interventricular septal thickness (IVSd, 1.90 ± 0.04 mm vs. 1.58 ± 0.03 mm, p < 0.001) and left atrial dimension (LAD, 5.46 ± 0.09 mm vs. 4.46 ± 0.12 mm, p < 0.001), and a reduction in ejection fraction (EF, 59.30 ± 0.98% vs. 74.04 ± 1.27%, p < 0.001) and fractional shortening (FS, 34.89 ± 1.30% vs. 44.48 ± 1.16%, p < 0.001) in ISO-induced cardiomyopathy group compared with the Control group. At week 10, EF (62.01 ± 1.87% vs. 52.60 ± 2.39%, p = 0.002) and FS (35.87 ± 1.51% vs. 29.78 ± 2.31%, p = 0.016) were increased in RDN group compared with Sham group, while IVSd (1.67 ± 0.03 mm vs. 1.54 ± 0.03 mm, p = 0.005) in RDN group was significantly thicker than in the Sham group. Surprisingly, comparing RDN group at week 10 with that of week 5, LAD (4.75 ± 0.25 mm vs. 5.41 ± 0.16 mm, p = 0.042) was significantly reduced (Table 1). Also, the plasma level of B-type natriuretic peptide (BNP) was significantly decreased in RDN group compared with Sham group (Fig. 1).

### Fibrosis of the LA, LV and kidney

**Figure 2:**
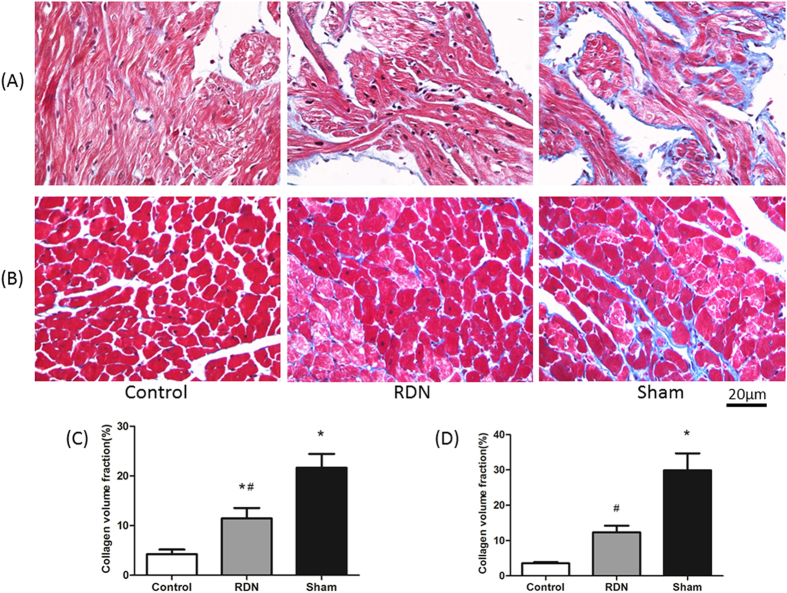
RDN Significantly Reduced CVF in Left Atrium and Ventricle Tissue. Histological section of left atrial (**A**) and left ventricular (**B**). Masson’s Trichrome Staining (magnification × 400), the blue stands for fibrosis. Comparison of collagen volume fraction (CVF,%) of left atrial (**C**) and left ventricular (**D**). *P < 0.05, RDN or Sham vs. Control; #P < 0.05, RDN group vs. Sham group (n = 6 per group).

**Figure 3:**
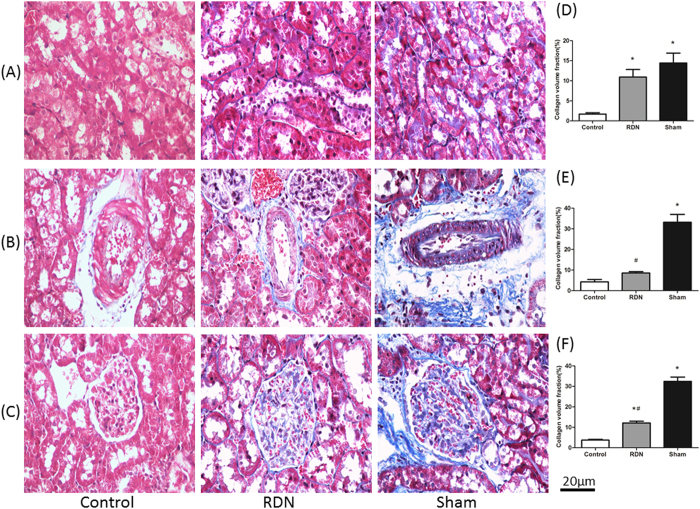
RDN Significantly Reduced CVF in Kidney. Histological section of renal trubulointerstitial (**A**), renal perivascular (**B**) and glomerular (**C**). Masson’s Trichrome Staining (magnification × 400), the blue stands for fibrosis. Comparison of collagen volume fraction (CVF,%) of trubulointerstitial (**D**), renal perivascular (**E**) and glomerular (**F**). *P < 0.05, RDN or Sham vs. Control; #P < 0.05, RDN group vs. Sham group (n = 6 per group).

CVF, a critical method to assess organic fibrosis, was evaluated in Masson’s Trichrome Staining sections. Compared with Sham group, the CVF of LA (11.44 ± 2.08% vs. 21.67 ± 2.77%, p = 0.003) and LV (12.32 ± 1.91% vs. 29.84 ± 4.84%, p < 0.01) were significantly decreased by RDN (Fig. 2). Interestingly, RDN treatment also had beneficial effects on renal glomerular (12.14 ± 0.84% vs. 32.42 ± 2.14%, p < 0.01) and perivascular fibrosis (8.59 ± 0.66% vs. 33.21 ± 3.82%, p < 0.01) (Fig. 3).

### Plasma Fibrosis Markers

**Figure 4:**
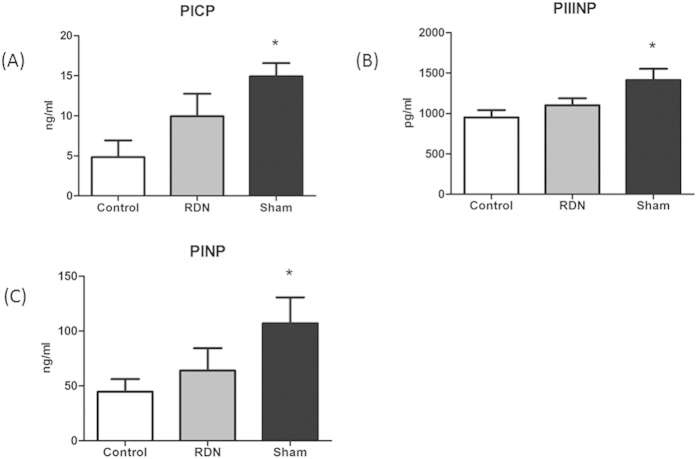
RDN Significantly Decreased Plasma Levels of PICP (**A**), PIIINP (**B**) and PINP (**C**). *P < 0.05, RDN or Sham vs. Control; #P < 0.05, RDN group vs. Sham group (n = 6 per group). PICP, carboxyl-terminal pro-peptide of type I collagen; PINP and PIIINP, amino-terminal pro-peptides of types I and III collagen.

The plasma levels of PINP, PIIINP and PICP reflect collagen synthesis. Compared with Control, PICP (14.94 ± 1.63 ng/ml vs. 4.85 ± 2.08 ng/ml, p < 0.01), PIIINP (1413.69 ± 139.17 pg/ml vs. 952.64 ± 90.63 pg/ml, p < 0.01) and PINP (107.03 ± 23.65 ng/ml vs. 44.71 ± 11.46 ng/ml, p = 0.037) were significantly increased in Sham group, which indicated the hyperactivity of collagen synthesis. However, this over-synthesis state was inhibited in RDN group (Fig. 4).

### RDN Rebalances RAAS Axis and Reduces SNS Activity

**Figure 5:**
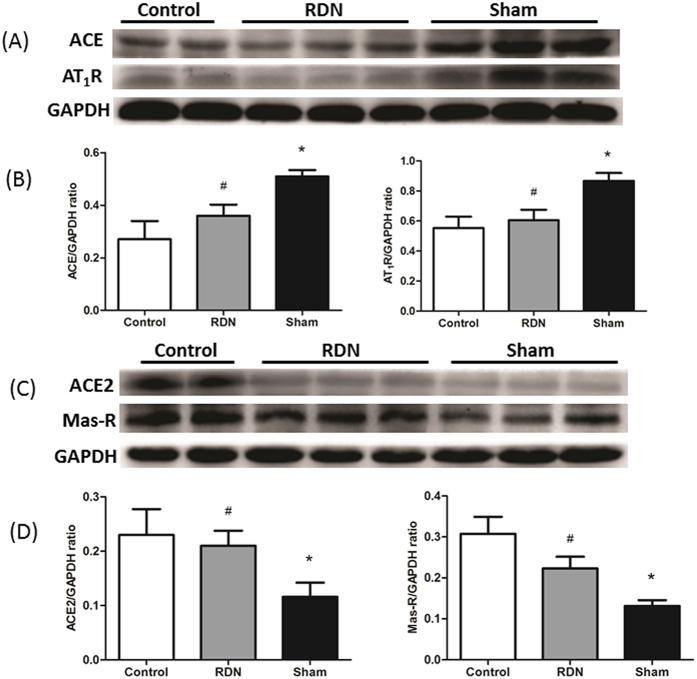
RDN Regulated Down ACE & AT_1_R (**A**) and Up ACE2 & Mas-R (**C**) in LV Tissue Protein Expression. RDN could significantly reduce ACE and AT_1_R protein expression (**B**), and increase ACE2 and Mas-R protein expression (**D**) compared with sham group. ^*^P < 0.05, RDN or Sham group vs. Control; ^#^P < 0.05, RDN group vs. Sham group (n = 6 per group). ACE, angiotensin-converting enzyme; AT_1_R, angiotensin II type-1 receptor; Mas-R, Mas-Receptor; RAAS, Renin–angiotensin–aldosterone system.

**Figure 6:**
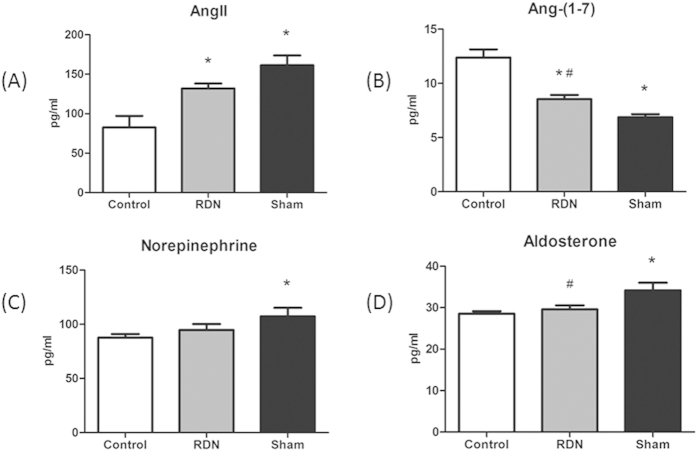
RDN Suppressed SNS Activity and Rebalanced RAAS Axis. Effects of RDN on the changes of plasma Ang II (**A**), Ang-(1-7) (**B**), noradrenaline (**C**), aldosterone (**D**) levels in the three groups. *P < 0.05, RDN or Sham vs. Control; #P < 0.05, RDN group vs. Sham group (n = 6 per group). Ang II, angiotensin II; RAAS, Renin–angiotensin–aldosterone system.

Compared with Sham group, the protective axis ACE2/Ang-(1-7)/Mas-R was significantly upregulated, while the exciting axis ACE/Ang II/AT_1_R was downregulated by RDN. ALD (29.57 ± 0.97 pg/ml vs. 34.2 ± 1.83 pg/ml, p < 0.01), a member of RAAS, was also significantly reduced. Plasma NE (107.35 ± 7.82 pg/ml vs. 87.66 ± 3.23 pg/ml, p < 0.05) was significantly increased in Sham group compared with Control. RDN alleviated the release of NE (Figs 5 and 6).

### Fibrosis-related Factors

**Figure 7:**
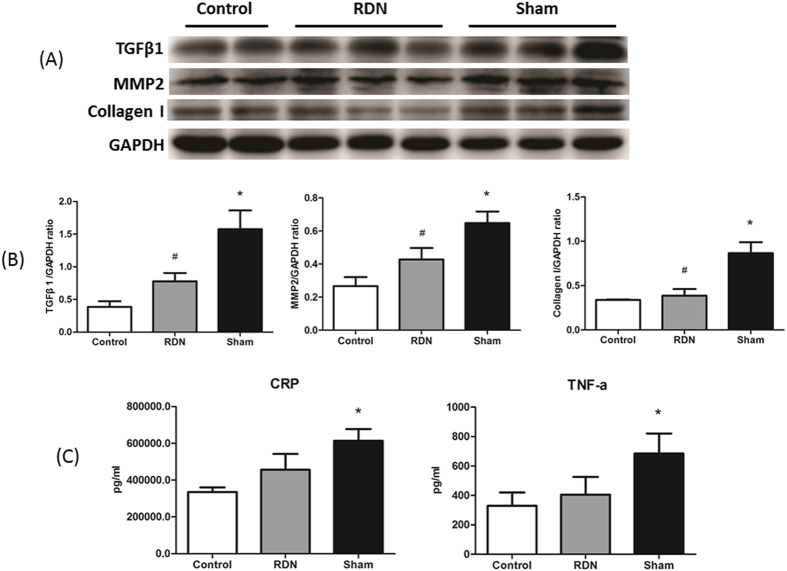
RDN Effect on Pro-Fibrosis Factors and Inflammatory Cytokines in LV Tissue and Plasma. The protein expression of TGFβ1, MMP2 and Collagen I (**A**). RDN significantly reduce TGFβ1, MMP2 and Collagen I protein expression (**B**) compared with Sham group. Effects of RDN on the changes of plasma CRP and TNF-α (**C**). ^*^P < 0.05, RDN or Sham group vs. Control; ^#^P < 0.05, RDN group vs. Sham group (n = 6 per group). TGFβ1, Transforming growth factor-β1; MMP2, matrix metalloproteinase 2; CRP, C-reactive protein; TNF-α, tumor necrosis factor α.

TGF-β1 and MMP2 are well-known fibrosis-related factors. Protein expression of TGF-β1 and MMP2 in LV was significantly decreased in RDN group compared with Sham group. Importantly, compared with Sham group, RDN significantly attenuated LV Collagen I protein expression (Fig. 7).

### Inflammatory Factors

Plasma inflammatory cytokines, such as TNF-α (684.74 ± 136.77 pg/ml vs. 329.52 ± 90.30 pg/ml, p < 0.05) and CRP (613615.65 ± 64478.81 pg/ml vs. 335631.60 ± 25138.63 pg/ml, p < 0.05), were significantly elevated in Sham group compared with Control, while decreasing tendency of these inflammatory factors were observed in RDN group, compared with the Sham (Fig. 7).

## Discussion

We firstly demonstrated that RDN has comprehensive anti-fibrosis effects on heart and kidneys. The main findings of our study were that: 1) RDN significantly decreased the hyperactivity of SNS and modulated the out-of-balanced RAAS axis, which related to the expression of TGF-β1 (core mechanism for organ fibrosis) in tissue. 2) RDN lowered valid collagen synthesis biomarkers concentration (PICP, PINP, PIIINP) and reduced cardio-renal CVF.

ISO-induced cardiomyopathy is a process from cardiac compensatory hypertrophy to decompensate heart failure, which is accompanied by dysfunction of kidney^18^,^19^. After continuous injection of ISO for 5 weeks, cardiac dysfunction and myocardial hypertrophy were verified by echocardiography. Meanwhile, LAD was significantly enlarged. At week10, we observed a further progression of cardiac dysfunction, EF and IVSd significantly decreased and BNP level significantly increased, associated with increased plasma NE levels, fibrogenic factor expression, increased inflammatory cytokines production, out-of-balanced RAAS axis, and development of left atrioventricular and renal fibrosis, and all of these changes were reversed by RDN.

Fibrosis is a common pathway to organ injury and failure^1^. A 3% increase in the CVF of fibrous tissue is associated with a 50% increase in the risk of adverse cardiovascular events^20^. Thus, a need clearly exists to find a way to slow, arrest, or even reverse the progression of tissue fibrogenesis. We and others have shown that RDN could prevent the progressive ventricular fibrosis with accompanying reduction in SNS activity^17^,^21^. In line with previous findings, we found that RDN significantly reduced the CVF of LV, simultaneously, we also provided evidence that ISO-induced cardiomyopathy caused a fairly striking increase in CVF in the LA and kidney, and that those were prevented by RDN. However, what underlie the beneficial effects by RDN were poorly defined.

Our present study defines some possible mechanisms by which RDN affects cardio-renal fibrosis process. Firstly, RDN inhibits excessive activation of SNS and modulates RAAS axis. Previous studies showed that inhibitors of SNS, such as beta-blockers, could attenuate and reverse remodeling in hypertension, heart failure and myocardial infarction^22^,^23^,^24^. As in our prior study, we found that RDN could regulate the expression of β1,2-receptors, which could contribute to improvements of fibrosis in myocardium^17^. What’s more, several studies pointed toward a relevant role of increased sympathetic activation for structural and functional changes in the kidney^25^. In accordance with these studies, RDN clearly reduced NE level down to Control group levels and prevented the progressive LA, LV and kidney damage. The RAAS is classically defined as a coordinated hormonal cascade in the control of multiple organs, such as cardiovascular and renal functions, mainly through the actions of Ang II^26^. Data from prior studies suggest that the inhibitors of ACE/Ang II/AT_1_R axis have beneficial effects on remodeling. Long-term treatment with AT_1_R antagonists could significantly decrease cardiac fibrosis and improve cardiac function^27^. Moreover, combined ACEI and ARB treatments in chronic heart failure could further prevent cardiac fibrosis, compared with ACEI or ARB alone^28^. Ang-(1-7) is mainly formed from Ang II or Ang I by ACE2^29^, which is part of the RAAS, with the counteracting effects against the adverse actions of Ang II. Ang-(1-7) can induce anti-fibrosis, vasodilatation, anti-proliferation and anti-hypertrophy via binding to the Mas-R^30^. The activation of ACE2/Ang-(1–7)/Mas-R axis has an inhibitive effect on pathological collagen synthesis. ACE2 can inhibit pathological hypertrophy, cardiac remodeling, and improve cardiac function^31^. Lakshmanan AP *et al.* also reported that telmisartan (ARB) attenuated renal fibrosis and oxidative stress through alteration of Mas-R expression^32^. On the molecular level, Ang II and ALD are major stimuli for increasing inflammation cytokines and TGF-β1, which promote fibroblast proliferation, migration, extracellular matrix remodeling and deposition of pro-collagen^33^,^34^. While, Ang-(1-7) decrease TGF-β1 mediated signalling in fibroblasts^35^. A critical finding in prior studies is that Ang-(1-7) axis provide a renoprotective role in the kidney diseases, administration of either an Ang-(1-7) antagonist or an ACE2 inhibitor aggravated the process of CKD^36^,^37^, while Ang-(1-7) reversed the damage of glomerular in rats^38^. In ISO-induced cardiomyopathy rats, we found that the downregulated ACE2/Ang-(1-7)/Mas-R axis produced a RAAS imbalance leading to abnormal predominance of the ACE/Ang II/AT_1_R axis. Consequently, these rats presented cardio-renal fibrosis and inflammation. Interestingly, RDN downregulated ACE/Ang II/AT_1_R axis and upregulated ACE2/Ang-(1-7)/Mas-R axis in our study. As we know, ALD can induce cardio-renal injury, inflammation, and fibrosis, long-term ALD exposure aggravates heart and kidney remodeling^33^,^39^. While, ALD antagonist can significantly attenuate remodeling of heart and kidney^40^,^41^. After 5 weeks therapy of RDN, the plasma levels of ALD was significantly decreased. Secondly, RDN significantly reduces pro-fibrosis factors and inflammation cytokines production. Abundant experimental evidence supports the notion that TGF-β1 plays an important role in the pathogenesis of fibrosis^1^,^34^. Simultaneously, TGF-β1 can upregulate mRNA level of MMP2 in cardiac fibroblasts, and the elevated protein expression of MMP2 could promote the activation of fibroblasts^42^, further increasing the collagen synthesis. Fumitaka Kuwahara *et al.* had reported that anti-TGF-β1 neutralizing antibody can inhibit fibroblast activation and subsequently prevented collagen mRNA induction and myocardial fibrosis^43^. In our study, RDN significantly reduced the expression of TGF-β1, MMP2 and Collagen I in LV tissue. Inflammatory cytokines, including TNF-α and CRP, are independently associated with increased cardiovascular and renal remodeling^44^,^45^. It has reported that TNF-α antagonism can attenuate the inflammation and cardiac fibrosis^46^. Trends toward decreased TNF-α and CRP after RDN were observed. Finally, RDN suppressed fiber synthesis. PINP, PIIINP and PICP are specific biomarkers for collagen synthesis^47^. Increased contents of PICP, PINP, and PIIINP in plasma suggest excessive collagen production and are highly associated with repair and fibrosis^48^. In recent experimental studies, RDN could remarkably depress cardiovascular ECM turnover and deposition, reflected by the three biomarkers^47^. In our study, these biomarkers sustained at an over-synthesis state in Sham group, RDN blocked this pathological state.

Hyperactivity of SNS and RAAS are common pathological processes in multiple organ remodeling. For example, overactivity of neurohumor becomes part of the disease process itself resulting in further worsening of cardio-renal function^49^,^50^. RDN reduce SNS activity, together with normalization of RAAS axis, might underlie the beneficial effects of RDN in regulating homeostasis, further reduce inflammatory cytokines and profibrogenic factors production, and then significantly decrease collagen synthesis.

In conclusions, RDN has the comprehensive suppression effects of cardiac and renal fibrosis in ISO-induced cardiomyopathy. RDN inhibits cardio-renal fibrogenesis through multiple pathways, including reduced SNS over-activity, rebalanced RAAS axis, and suppressed actions of TGF-β1.

## Additional Information

**How to cite this article**: Liu, Q. *et al.* Renal Denervation Findings on Cardiac and Renal Fibrosis in Rats with Isoproterenol Induced Cardiomyopathy. *Sci. Rep.* **5**, 18582; doi: 10.1038/srep18582 (2015).

## Figures and Tables

**Table 1:** Echocardiography parameters in 5 and 10 week.

Group	week	EF(%)	FS(%)	IVSd(mm)	LAD(mm)
Control	5	74.04 ± 1.27	44.48 ± 1.16	1.58 ± 0.03	4.46 ± 0.12
	10	73.65 ± 0.79	44.07 ± 0.72	1.64 ± 0.02	4.29 ± 0.06
RDN	5	59.19 ± 1.54*	33.58 ± 1.48*	1.89 ± 0.04*	5.41 ± 0.16*
	10	62.01 ± 1.87^†^	35.87 ± 1.51^†^	1.67 ± 0.03^#†^	4.75 ± 0.25^#^
Sham	5	59.43 ± 1.20*	36.64 ± 2.25*	1.92 ± 0.09*	5.52 ± 0.08*
	10	52.60 ± 2.39^#^	29.78 ± 2.31	1.54 ± 0.03^#^	5.19 ± 0.26

^*^P < 0.05, RDN or Sham group vs. Control at 5 week; ^†^P < 0.05 RDN group vs. Sham group at 10 week; ^#^P < 0.05, 10 week vs. 5 week.
